# Measuring light transport properties using speckle patterns as structured illumination

**DOI:** 10.1038/s41598-019-47256-8

**Published:** 2019-08-01

**Authors:** Pranay Jain, Sanjay E. Sarma

**Affiliations:** Field Intelligence Laboratory, Department of Mechanical Engineering, Massachusetts Institute of Technology, Cambridge, MA 02139 USA

**Keywords:** Optical sensors, Biomedical engineering, Imaging techniques, Imaging and sensing, Spectrophotometry

## Abstract

The measurement of light absorption and scattering properties of biological materials has several diagnostic and therapeutic applications. We can measure these properties for skin without contact using structured illumination and imaging. However, building simple handheld devices remains challenging due to motion artefacts and moving targets. To overcome this limitation, we project random speckle patterns instead of discrete spatial frequencies on the target. Since random patterns are spatially broadband, they capture more information per image, enabling frame-by-frame analysis. In this paper, we describe the statistics of objective speckles and demonstrate how the optical system is designed for spatially bandlimited illumination. Next, we use a diverse set of liquid tissue phantom to validate the method. We successfully demonstrate that a calibrated instrument can independently predict the two primary light transport properties of a homogeneous turbid system. This work is a starting point for analysing skin and other heterogeneous biological media in the future.

## Introduction

Many common materials are optically turbid in nature. They are neither entirely transparent nor opaque. Light enters these media and diffuses, making them look *cloudy*. A prominent example is skin. When we illuminate a small spot on skin, adjoining regions also appear to glow because of optical diffusion. This behaviour is quantified as the material’s light transport properties, principally the absorption coefficient *μ*_*a*_ and the reduced scattering coefficient ${\mu ^{\prime} }_{s}$^1^. Measuring these properties is useful in several applications. In biological materials, this helps us distinguish and characterize internal structures and pigments otherwise hidden from the naked eye^2,3^. This information is vital for accurate diagnosis and therapy of skin-related conditions like melanoma, lesions and burns^4^. In general, there are applications beyond medicine as well. In foods like milk and cheese, optical properties allow us to estimate the size distribution and concentration of suspended fats and proteins based on their different scattering properties^5^. In dispersions like moisturizers and paints, this allows to estimate homogeneity and colloidal stability.

Existing laboratory-scale methods can accurately measure light transport properties of turbid media, but usually require significant sample preparation and dilution^6^. There is an increasing interest in *in-vivo* methods that allow measurement without damaging the sample, particularly for applications in medicine^7^. *In-vivo* methods essentially employ a system identification approach, where the samples are illuminated with visible or infrared radiation and the backscatter is measured. Comparing the illumination and backscatter in time or in space lets us estimate the light transport properties. Most commercial *in-vivo* methods use fibre-optic probes and perform time-domain analysis^8^, but struggle to find a place in routine clinical use because of poor spatial resolution. We are interested in using imaging for *in-vivo* analysis in order to overcome the spatial resolution limitation of fibre bundles. Imaging-based methods are relatively new and increasingly gaining acceptance^9^. In addition to improved spatial resolution, they allow us to work with compact optical hardware, offloading much of the burden to software and computation. They are therefore promising for rapid, non-invasive, and even non-contact analysis in several medical or industrial applications.

**Figure 1:**
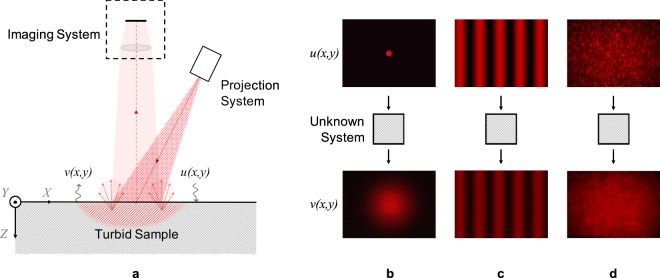
(**a**) Intensity distribution input *u*(*x*, *y*) and output *v*(*x*, *y*) for system identification by backscatter imaging: (**b**) Narrow beam as input and PSF as output. (**c**) Structured illumination with discrete spatial frequency as input and amplitude attenuated output. (**d**) Broadband speckle pattern as input and spatially low-pass filtered output.

In imaging-based methods (Fig. 1a), we project a monochromatic pattern with a spatial intensity distribution *u*(*x*, *y*) on the sample surface and observe the backscatter distribution *v*(*x*, *y*) using an image sensor. Output *v*(*x*, *y*) is different from input *u*(*x*, *y*) because instead of simply reflecting off the surface, light enters the medium and diffuses before being backscattered. The output is therefore a blurred version of the input, or in other words, the output is a spatially low-pass filtered version of the input. In the most straightforward approach, we can project a narrow orthogonal beam of light on the sample and directly observe the Point Spread Function (PSF) or the impulse response^10^ (Fig. 1b). Alternatively, we can use *structured illumination*, projecting patterns of discrete spatial frequencies using micromirror devices or diffraction gratings and observing the medium’s response in the spatial frequency domain^9^ (Fig. 1c). While these methods are identical in theory, their measurement accuracy differs in practice due to different sensitivity to image noise.

Image sensors let us observe backscatter at a much finer spatial resolution than fibre optic probes, collecting millions of 8-bit to 16-bit resolution pixels per observation. However, image sensors also come with lower dynamic range compared to other photodetectors^11^, adding a source of uncertainty in the estimated properties. When we project an impulse, the observed PSF is limited to a small region around the illuminated spot, and only a few pixels have a distinguishable signal. We effectively waste a majority of the pixel resources, and eventually limit the sensitivity and the precision of the instrument. Using structured illumination overcomes this challenge by distributing the backscatter signal over the entire image plane. The illumination can be controlled such that light intensity signal at all pixels is well within the range of the sensor. The instrument can therefore compensate for poor SNR with the millions of data points.

In essence, structured illumination helps us trade-off measurement uncertainty for pixel redundancy. As shown in Fig. 1c, we project light in a single spatial frequency on the sample and estimate the amplitude attenuation in backscatter. By extracting the sinusoid at the known spatial frequency from the backscatter image, we obtain the spatial frequency response and reject the uncorrelated noise in individual pixel readings. While we collect millions of noisy pixel readings, we reduce them to a single high precision data point. We therefore require observations at multiple spatial frequencies to reasonably estimate the sample’s light transport properties. The turbid sample and instrument need to be stationary during these multiple observations. This can prove to be a challenge for hand-held devices or with rapidly moving or changing turbid media.

In this paper, we use *objective speckle patterns* as structured illumination (Fig. 1d) instead of discrete sinusoids. In discrete sinusoids, the signal energy is concentrated in a sharply narrow band of spatial frequencies. In contrast, speckle patterns are randomly distributed in space, distributing the signal energy continuously over a broader spatial frequency band. This allows us to simultaneously observe the sample’s response over a range of spatial frequencies, even with a single observation. Ultimately this enables us measure more rapidly, allowing us to eliminate motion artefacts between captured frames. By changing the nature of illumination, we are effectively changing the trade-off between measurement uncertainty and pixel redundancy. Projecting discrete sinusoids maximises redundancy at the cost of data and time. On the other hand, projecting ideal white noise maximizes speed at the cost of instrument precision. Since speckle patterns can be adapted to control their spatial frequency content, they effectively provide an additional degree of freedom to the imaging-based methods. The system can therefore be designed based on application-specific preferences for speed or precision, while maintaining the merit of structured illumination in using all the pixel resources judicially.

In the following sections, we first discuss the statistics of objective speckle patterns as spatially random structured illumination. We experimentally demonstrate how speckle projection may be adapted to desired bandlimited illumination. Next, we describe the system identification approach in the proposed imaging-based method. We present results from experimental data collected using multiple liquid tissue phantoms at 635 nm illumination. The phantoms are prepared by mixing different relative concentrations of Intralipid^TM^ 20%, methylene blue and deionized water. We compare the experimental results to predictions using optical diffusion simulations. Finally, we discuss opportunities to use spatially-random structured illumination for examining complex and heterogeneous biological materials, and eventually to develop devices for use in diagnostic and therapeutic applications.

## Speckle Pattern Statistics

When we point an expanded laser beam at almost any surface other than a polished mirror, we readily see a randomly-distributed high-contrast speckle pattern overlapping the illuminated region. These speckles find utility in applications like displacement measurement^12^, surface roughness estimation^13^, and more recently blood perfusion analysis^14^. Speckles can also act as undesirable multiplicative noise in coherent imaging techniques like ultrasound^15^ or radar imaging^16^ and require suppression there. In the present method, we are using speckle patterns as structured illumination precisely for their inherently random nature and the high contrast.

**Figure 2:**
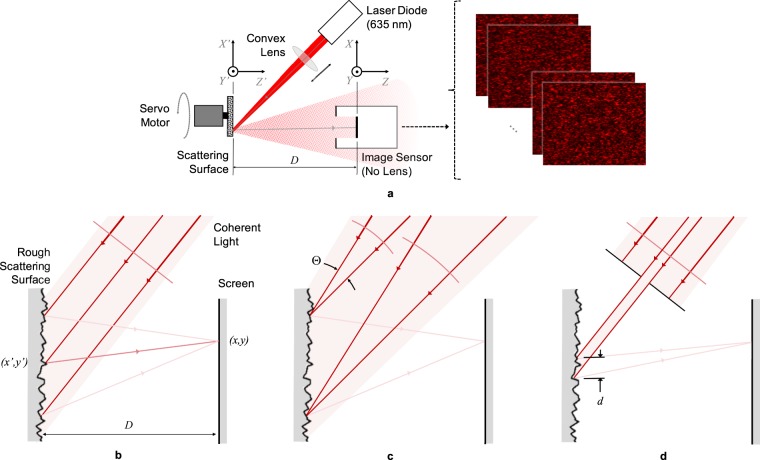
(**a**) Schematic of the method used to observe objective speckle patterns directly on an image sensor. (**b**) A collimated beam and a wide spot size lead to fully-developed speckles. (**c**) A converging or diverging defocussed beam or (**d**) a small spot size lead to partially-developed speckles.

Speckles form due to interference when coherent light scatters off non-uniform media. When we illuminate a rough surface, we find that the scattered light is riddled with speckles in 3-dimensional space. We can observe a slice of this 3D pattern by simply placing a screen or an image sensor in the path of the scattered light (Fig. 2a). This projected slice is known as an *objective* speckle pattern. In the present method (Fig. 1d), we replace the screen with the turbid sample, effectively illuminating a sample surface with objective speckles. The intensity distribution *u*(*x*, *y*) of the projected pattern is equivalent to a 2D random signal, and more accurately, a realization of a 2D random process. Since the random process serves as the input for system identification, we care about its stationarity and ergodicity, and its first and second-order statistics.

### Fully and Partially Developed Speckles

Speckle patterns are interferometric phenomena. The electric field amplitude at any point on the screen is a sum of all coherent wavefronts that originate from illuminated points on the scattering surface (Fig. 2a). Using far-field approximations, we can reduce the relation to a Fourier transform^17^. The intensity *u*(*x*, *y*) is proportional to the square of the electric field amplitude $\underline{E}(x,y)$, and hence approximately proportional to the square of the Fourier transform of the electric field amplitude ${\underline{E}}_{s}(x^{\prime} ,y^{\prime} )$ at the scattering surface:

1 $$u(x,y)\mathop{\propto }\limits_{ \sim }{| {\mathcal F} [{\underline{E}}_{s}(x^{\prime} ,y^{\prime} )]|}^{2}$$

As coherent light scatters, its phase $\angle {\underline{E}}_{s}(x^{\prime} ,y^{\prime} )$ varies randomly because of the path length difference introduced by the randomly varying profile of the rough scattering surface. If the surface profile is an ideal 2D white noise process in the plane of the scattering surface, the speckle pattern on the parallel screen will also be a 2D white noise process. The analysis and assumptions explaining this are included in the accompanying Supplementary Material. The size of speckle grains for such a pattern would be infinitely small, although diffraction-limited by the wavelength *λ* of incident light. Such a speckle pattern, with a vanishing specular field, is known to be *fully-developed*. If we assume that speckle patterns are stationary and ergodic, statistics of the intensity *u*(*x*, *y*) at any point on the screen is the same as that at all other points. For fully-developed speckles, the Probability Density Function (PDF) of intensity *u* is a *χ*^2^ distribution with 2 degrees of freedom. Despite the non-uniform or non-Gaussian distribution of *u*, the random process is still white, implying that the power spectrum in the spatial frequency domain is flat, although only up to $|\overrightarrow{k}|\sim 1/\lambda$.

In practice, fully-developed speckle patterns are difficult to generate due to the detailed structure of the scattering surface and the finite aperture of the optical system^18^. Additionally, they are difficult to observe by imaging because of the point spread function and finite spatial resolution of imaging optics. Therefore, speckle patterns are usually found to have finite speckle grain sizes greater than *λ*. Such speckles are known to be *partially-developed*. If the incident radiation is defocussed (Fig. 2b), similar to that in Köhler Illumination, the projected speckles are convolved with the corresponding blur disk. If a circular defocussed beam converges at a small angle Θ, the fully-developed speckles are convolved with a circle of diameter Θ*D*, where *D* is the spacing between the scattering surface and the screen. The result is a finite speckle size of the order of Θ*D*. A similar effect is recorded due to a small illumination spot size on the scattering surface (Fig. 2c). Illuminating only a circular spot of diameter *d* is equivalent to multiplying ${\underline{E}}_{s}(x^{\prime} ,y^{\prime} )$ in Equation (1) with a circular aperture of the same diameter. The Fourier transform is therefore convolved with the corresponding diffraction pattern, Airy disc for a circular aperture. The result is a partially-developed speckle pattern with speckle size of the order of *λD*/*d*.

The convolution that leads to partially-developed speckles also changes its first and second-order statistics. The convolution is effectively a weighted average of neighbouring random variables. The PDF of intensity *u* is therefore a *χ*^2^ distribution with degrees of freedom higher than 2. This is expected to undesirably reduce the standard deviation, and hence the input signal power in system identification. Further, the power spectrum is bandlimited due to the spatial filtering. The latter is usually desirable in order to prevent image aliasing during observation, and to maximize signal power in the spatial frequency band of interest. We can therefore design the aperture size of our optical system to project an input random signal *u*(*x*, *y*) with suitable statistics of the projected pattern, especially the power spectrum band.

### Effect of Aperture Size

The far-field approximations and the ergodicity and stationarity assumptions simplify the above analysis for an intuitive explanation of speckle phenomena. It serves the purpose of identifying aperture size as a tuneable parameter in system design. However, the analytical solutions may deviate from the observed physical nature of speckles. To bridge this gap, we experimentally verify the effect of spot size or aperture size on first and second-order statistics of speckles.

Figure 2a illustrates the optical breadboard setup we use to observe speckle patterns at different apertures. A 635 nm 4.5 mW laser diode emits a collimated and coherent beam with a 5.0 mm × 1.9 mm elliptical cross-section. A convex lens converges the beam, creating an illuminated spot on a piece of card stock (*X*′*Y*′ plane). Light scatters off the rough card stock surface and forms objective speckles directly on an image sensor (*XY* plane) placed parallel to the card, at a distance *D* = 86 mm. The monochromatic CMOS image sensor has 1280 × 1024 8-bit pixels in a uniform grid with 4.8 μm pitch. We therefore observe a 6.1 mm × 4.9 mm speckle pattern. For simplicity, we persist with the *u*(*x*, *y*) notation despite the discrete sampling when imaging.

**Figure 3:**
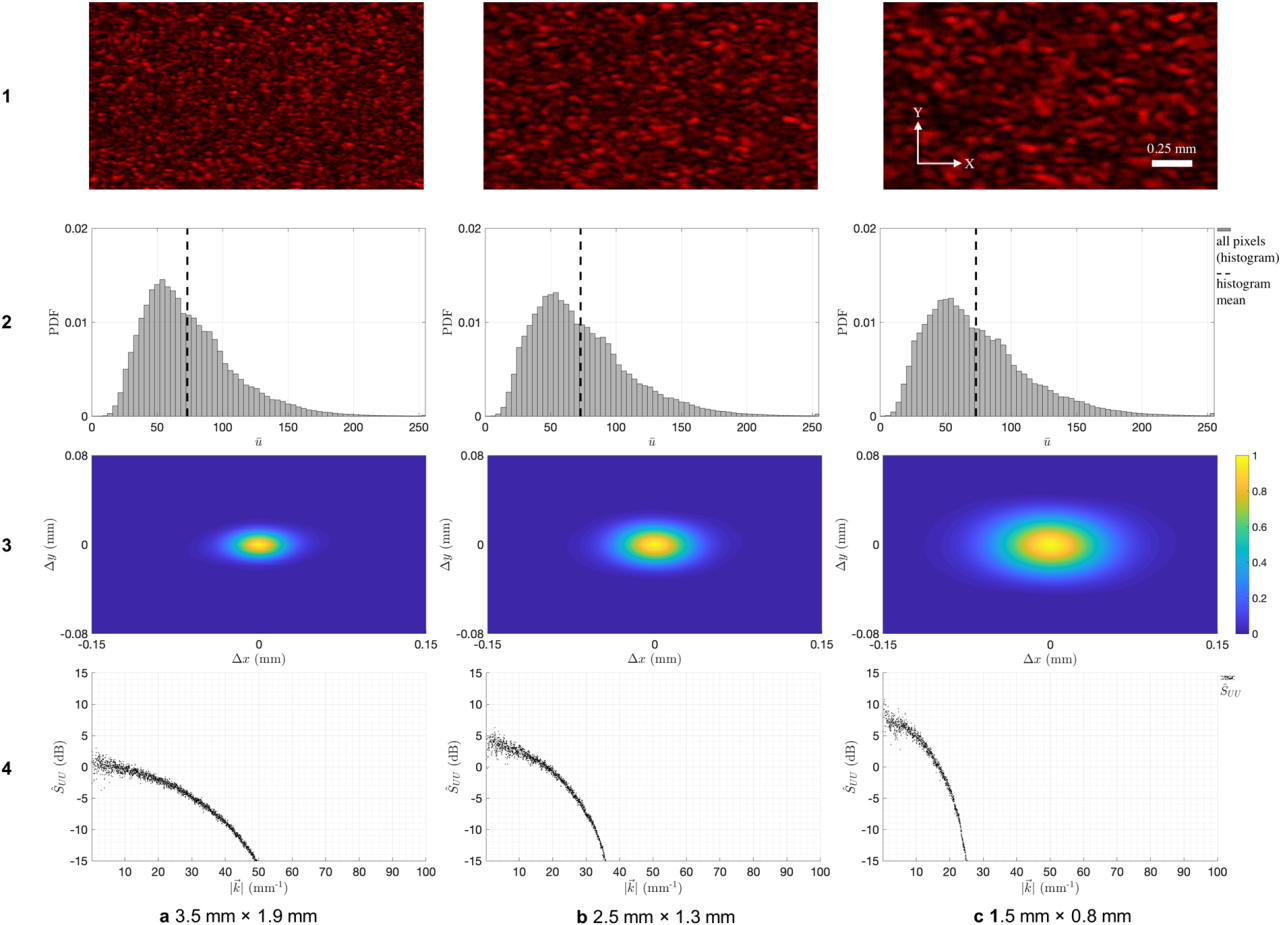
Statistics of speckle patterns observed at three (**a** to **c**) elliptical aperture sizes: (**1**) Sample speckle image. First-order statistics: (**2**) histogram of all pixel readings. Second-order statistics: (**3**) estimated ACF ${\hat{R}}_{UU}({\rm{\Delta }}x,{\rm{\Delta }}y)$ and (**4**) estimated PSD ${\hat{S}}_{UU}(0,{k}_{y})$.

We mount the card stock on a servo motor perpendicular to its axis of rotation. Rotating the card in the *X*′*Y*′ plane allows us to change the illuminated location on the card without modifying any optical parameters. This lets us observe multiple speckle patterns *u*_*i*_(*x*, *y*); *i* = 1 … *N*, where each pattern is an independent realization of the same random process *U*(*x*, *y*). We capture *N* = 10 patterns by moving the servo randomly between each frame. This lets us increase the sampled data without using larger image sensors. We repeat the above steps for three elliptical aperture sizes: 3.5 mm × 1.9 mm, 2.5 mm × 1.3 mm, and 1.5 mm × 0.8 mm. The aperture sizes are adjusted by moving the convex lens axially to scale the spot size on *X*′*Y*′ plane. For illustration, row 1 of Fig. 3a–c shows a cropped part of the first observed speckle pattern for each configuration.

With the collected data, we confirm that the observed speckle pattern processes are at least Wide-Sense Stationary (WSS) and *ergodic in the mean*. To validate stationarity, we estimated the Auto-Correlation Function (ACF) ${\hat{R}}_{UU}({\rm{\Delta }}x,{\rm{\Delta }}y)$ of the process. As required, it decays rapidly and monotonically with increasing spatial delay. For ergodicity, we first confirmed that the *N* realizations are independent. For this, we computed the cross-correlations between all pairs and found them to be at least two orders of magnitude smaller than ACF, and hence negligible. We then computed the means at each pixel across *N* realizations, and compared them with the means at each realization across all pixels. The expected mean estimates from both coincide with reasonable certainty, satisfying the minimum requirement for ergodicity. These results are included with more details in the accompanying Supplementary Material.

The *first-order statistics* of a random process describe the probability distribution of all resulting random variables. Row 2 of Fig. 3a–c gives the normalized distribution for all observed pixel readings in the three configurations. Since the speckles are stationary and ergodic, the distribution is independent of (*x*, *y*) pixel position. While the distribution is arguably similar to a higher-order *χ*^2^ distribution, its order does not increase with decreasing aperture size. Although this is contrary to the above analysis, it is advantageous to the present method because no loss in standard deviation or signal energy is reported with decreasing aperture size.

The *second-order statistics* of a random process describe the relation between any two resulting random variables. Rows 3 and 4 of Fig. 3a–c give the normalized ACF estimate ${\hat{R}}_{UU}({\rm{\Delta }}x,{\rm{\Delta }}y)$ and Power Spectral Density (PSD) estimate ${\hat{S}}_{UU}({k}_{x},{k}_{y})$; *k*_*x*_ = 0 for the three configurations. Again, due to stationarity, these estimates are independent of (*x*, *y*) pixel position. The ACF has elliptical contours because of the elliptical shape of the aperture. Correspondingly, PSD is broader at *k*_*x*_ = 0 than at *k*_*y*_ = 0; only the former is shown for simplicity. As we decrease aperture size, ACF broadens and PSD narrows, also increasing signal power in the narrower spatial frequency band. This behaviour is as predicted by above analysis, confirming the effect of aperture size on spectral content of objective speckles.

## Method

**Figure 4:**
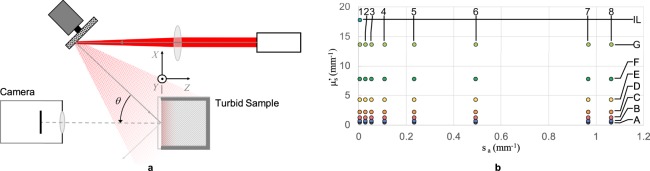
(**a**) Schematic of the method used to observe backscatter images for liquid tissue phantoms. (**b**) Reference light transport properties (*μ*_*a*_ and ${\mu ^{\prime} }_{s}$) at 635 nm of the 57 phantoms, as measured by Aernouts *et al*.^19^.

Figure 4a illustrates the setup we use to project objective speckles on a turbid sample and image the backscatter. We project speckles in the same way as shown earlier in Fig. 2a, using the same light source, optics and servo-mounted card. Here, instead of projecting objective speckles on an image sensor, we project them on the turbid sample under examination. For these experiments, we use liquid tissue phantoms contained in a 20 mm path length glass cuvette. Speckles are incident on a flat cuvette face (*XY* plane) at angle *θ* = 45° so that specular reflections from the glass surfaces don’t obscure the backscatter image. All other faces of the cuvette are painted black to minimize internal reflections. We image the backscatter using a monochromatic CMOS camera orthogonal to the cuvette face. We let the camera automatically adjust exposure time for darker images in order to make best use of its dynamic range. The pixel readings are divided by the exposure time offline prior to their use in image analysis and system identification. This ensures that all data has the same gain and approximately the same SNR.

The spatial resolving power of the imaging system is an important parameter in the current measurement. It limits the maximum spatial frequency we can observe without aliasing. Higher spatial frequencies in the projected speckle pattern can wrap and distort the observed spatial frequency content of a backscatter image. We use an image sensor with 1280 × 1024 8-bit pixels and *f*/4 focusing optics. The 34 mm × 25 mm cuvette face maps to 673 × 495 pixels on the image sensor. Here, the 4.8 μm × 4.8 μm pixels are bigger than the resolving power of the lens system based on the Rayleigh Criterion. Therefore, the Nyquist frequency of roughly 10 mm^−1^ determines the maximum observable spatial frequency. We accordingly limit the spatial frequency band of the projected pattern by tuning the aperture size of projection optics, as we discussed earlier. This tuning is more commonly described as setting a minimum size for individual speckles.

We validate the method by using it to evaluate tissue phantoms with known reference light transport properties. For this, we prepared 57 phantoms, using Intralipid^TM^ 20%, methylene blue and deionized water, that closely replicate those used by Aernouts *et al*.^19^ to measure light transport properties using a double integrating sphere setup. We use their phantom nomenclature (A1 … A8, …, G1 … G8, and IL) for consistency, and use their reported results at 635 nm as our reference (Fig. 4b). Phantoms with the same letter prefix have the same concentration of Intralipid^TM^ 20%, and hence approximately the same ${\mu ^{\prime} }_{s}$. Those with the same number suffix have the same concentration of methylene blue, and hence the same *μ*_*a*_. The phantom ‘IL’ is undiluted Intralipid^TM^ 20% with no dye. The phantoms represent a wide range of properties, especially suited for biological applications. For instance, skin tissue typically has light transport properties in the range^1^: ${\mu ^{\prime} }_{s}$ ∈ [1, 10] mm^−1^ and *μ*_*a*_ ∈ [0.003, 0.4] mm^−1^, and 30 of the 57 phantoms lie in it.

Same as earlier, we rotate the card to project *N* = 10 independent speckle patterns *u*_*i*_(*x*, *y*); *i* = 1 … *N* on the sample. Since we rotate the card randomly between observations, each *u*_*i*_(*x*, *y*) input is unique and no turbid phantom is examined with the same set of inputs. Despite their uniqueness, all *u*_*i*_(*x*, *y*) are realizations of the same random process *U*(*x*, *y*). Before introducing any liquid phantoms in the cuvette, we place a thin screen on the exposed window and collect *N* = 10 diffuse reflection images and use these to characterize the process *U*(*x*, *y*). Since the optical configuration is unchanged between measurements, we can use this prior reference measurement as the input random process for all phantoms. We actively image only the *N* = 10 backscatters *v*_*i*_(*x*, *y*) for each phantom and use them to characterize the corresponding output random process *V*(*x*, *y*). The system identification approach using these input and output processes is discussed later.

In practice, our ability to rely on a single set of prior reference measurement or calibration depends on the stability of speckle projection optics. This is easy to obtain in a laboratory setting, but not necessarily during field use. In that case, recording the input random process prior to a measurement will be cumbersome and not always possible. If observing the input simultaneously becomes imperative, it may be necessary to add a splitter and a second image sensor to the system.

### Model system response

When a narrow beam of light with unit power is incident at any point (*x*_0_, *y*_0_) on a turbid medium, the diffuse backscatter is the linear impulse response *h*(*x*, *y*; *x*_0_, *y*_0_). With structured illumination, the diffuse backscatter *v*(*x*, *y*) can therefore be described as a convolution of the impulse response with input *u*(*x*, *y*):

2 $$v(x,y)=(h\ast u)(x,y)$$

The phantoms used in the present experiment are stable emulsions and hence uniform. In the cuvette in Fig. 4a, they may be considered as semi-infinite if all dimensions are at least an order of magnitude greater than the photon mean free path *l* = 1/(*μ*_*a*_ + ${\mu ^{\prime} }_{s}$). This makes the impulse response independent of position (*x*_0_, *y*_0_) and reduces it to a radially symmetric function *h*(*r*), where *r* is the scalar distance from point (*x*_0_, *y*_0_). For system identification we are interested in modelling the sample’s spatial frequency response *H*(*k*_*x*_,*k*_*y*_), as we discuss in the next section. Since the impulse response is radially symmetric, the spatial frequency response also reduces to a 1D function *H*(*k*_*r*_).

The impulse and frequency responses of a uniform semi-infinite medium are independent of the nature of illumination used to measure the light transport properties. Cuccia *et al*.^9^ discusses model responses in the context of structured illumination with discrete spatial frequencies (Fig. 1c). We extend the same methods for random speckle illumination. We estimate the model impulse and frequency response for a turbid medium with a given set of light transport properties using White Monte Carlo (WMC) Ray Tracing simulations, as described by Jacques and Wang^20^, and Kienle and Patterson^21^. The predicted responses for a range of parameters (*μ*_*a*_, ${\mu ^{\prime} }_{s}$ and *g*) are included in the Supplementary Material.

**Figure 5:**
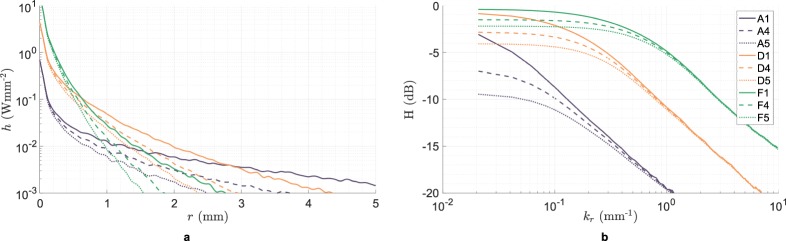
(**a**) Impulse response *h*(*r*) and (**b**) spatial frequency response *H*(*k*_*r*_) as predicted for nine liquid tissue phantoms using WMC simulations.

The model responses for 9 of the 57 phantoms are shown in Fig. 5a,b for illustration. For the purpose of estimating these responses, we use the reference *μ*_*a*_ and ${\mu ^{\prime} }_{s}$ values in Fig. 4b and we assume asymmetry factor *g* = 0.7. The response is not significantly sensitive to changes in *g* for a given reduced scattering coefficient ${\mu ^{\prime} }_{s}$. We therefore ignore its effect. Since we simulated only 10^5^ photons per sample to represent the ensemble, the impulse response estimates are noisy, but still distinguishable.

The predicted responses make intuitive sense. For turbid samples with the same *μ*_*a*_, incident photons go deeper into media with lower ${\mu ^{\prime} }_{s}$ and eventually escape radially further away from the origin. This widens the impulse response and narrows the frequency response. For a given input *u*(*x*, *y*), the output *v*(*x*, *y*) is therefore more blurred for such samples. In the other case, for samples with the same ${\mu ^{\prime} }_{s}$, diffusing photons face a larger probability of being absorbed in media with higher *μ*_*a*_. The photons with longer path lengths are more frequently absorbed. That is why the change in system response due to *μ*_*a*_ is more notable for samples with lower ${\mu ^{\prime} }_{s}$ than others. In the spatial frequency domain, the effect is akin to that of a negative feedback loop. Increasing *μ*_*a*_ decreases the gain while pushing the cutoff to higher spatial frequencies. The backscatter *v*(*x*, *y*) is therefore less bright, but also less blurred.

In summary, we can interpret that the impulse or frequency response of a sample is uniquely dependent on the underlying (*μ*_*a*_, ${\mu ^{\prime} }_{s}$) pair. Therefore, evaluating it is necessary and sufficient for estimating the medium’s two light transport properties.

### System identification

Our goal with system identification is to estimate the unknown light transport properties using our knowledge of the inputs and outputs. The intermediate step is to evaluate the system response *h*(*r*) or *H*(*k*_*r*_). For any given phantom, since the inputs *u*_*i*_(*x*, *y*) are realizations of a stationary random process *U*(*x*, *y*), and since the system is linear and spatially invariant, outputs *v*_*i*_(*x*, *y*) can also be described by another random process *V*(*x*, *y*). We can therefore restate eq. (2) as follows:

3 $${S}_{VV}({k}_{r})={|H({k}_{r})|}^{2}{S}_{UU}({k}_{r})$$

Here, *S*_*VV*_ and *S*_*UU*_ are the Power Spectral Densities (PSDs) of the input and output processes respectively. We estimate these from the *N* backscatter images captured for the sample and the *N* input reference images captured independently with the help of a temporary screen on cuvette window.

A challenge is to overcome the effect of edges and scratches on the glass surface, which can introduce unknown fixed patterns overlapping the observed *v*_*i*_(*x*, *y*) images, and need to be eliminated before we use eq. (3). We do this by subtracting the average cross-correlation between the *N*(*N* − 1) possible image pairs from the average auto-correlations of the *N* images:

4 $${\hat{R}}_{VV}(r)\iff {\hat{R}}_{VV}(x,y)=\frac{1}{N}\sum _{i=j}\,{R}_{{v}_{i}{v}_{j}}(x,y)-\frac{1}{N(N-\mathrm{1)}}\sum _{i\ne j}\,{R}_{{v}_{i}{v}_{j}}(x,y)$$

Here ${\hat{R}}_{VV}$ is the estimated auto-correlation of the isolated random process *V*(*x*, *y*). We take its Fourier transform to obtain the PSD estimate ${\hat{S}}_{VV}$ using Wiener-Khinchin theorem.

Another challenge is that PSD estimates of random processes are noisy by nature. We can minimize the noise by using windowing or other signal processing techniques, but cannot entirely eliminate it even in theory. Dividing two PSD estimates in eq. (3) makes the frequency response estimate even noisier. Instead of fitting model responses to noisy estimates, we reduce the estimated PSDs to two response parameters *H*_*DC*_ and *H*_*AC*_:

5 $${\rm{DC}}\,{\rm{Response}}:{H}_{DC}=\frac{{\hat{S}}_{VV}({k}_{r}\to \mathrm{0)}}{{\hat{S}}_{UU}({k}_{r}\to \mathrm{0)}}\approx {|H\mathrm{(0)}|}^{2}$$

6 $$\begin{array}{rcl}{\rm{AC}}\,{\rm{Response}}:{H}_{AC} & = & \frac{{\hat{S}}_{UU}({k}_{r}\to \mathrm{0)}}{{\hat{S}}_{VV}({k}_{r}\to \mathrm{0)}}\cdot \frac{\int {\hat{S}}_{VV}({k}_{r}\mathrm{)2}\pi {k}_{r}d{k}_{r}}{\int {\hat{S}}_{UU}({k}_{r}\mathrm{)2}\pi {k}_{r}d{k}_{r}}\\  & \approx  & \frac{1}{{|H\mathrm{(0)}|}^{2}}\cdot \frac{\int {|H({k}_{r})|}^{2}{\hat{S}}_{UU}({k}_{r}\mathrm{)2}\pi {k}_{r}d{k}_{r}}{\int {\hat{S}}_{UU}({k}_{r}\mathrm{)2}\pi {k}_{r}d{k}_{r}}\end{array}$$

*H*_*DC*_, the DC or low-frequency response, is a measure of the fraction of photons backscattered by the medium. *H*_*AC*_ is a measure of the blurring due to diffusion. It is the signal power or contrast retained by the backscatter, and is therefore normalized to unit DC response. While PSD estimates are noisy, their integrals are better behaved due to the uncorrelated nature of PSD noise. Both the parameters are ratios comparing the output to the input, and should be between 1 and 0. The left sides of eqs (5) and (6) are estimated from experimental data while the right sides are the corresponding measures in terms of the input and modelled system response.

## Results

**Figure 6:**
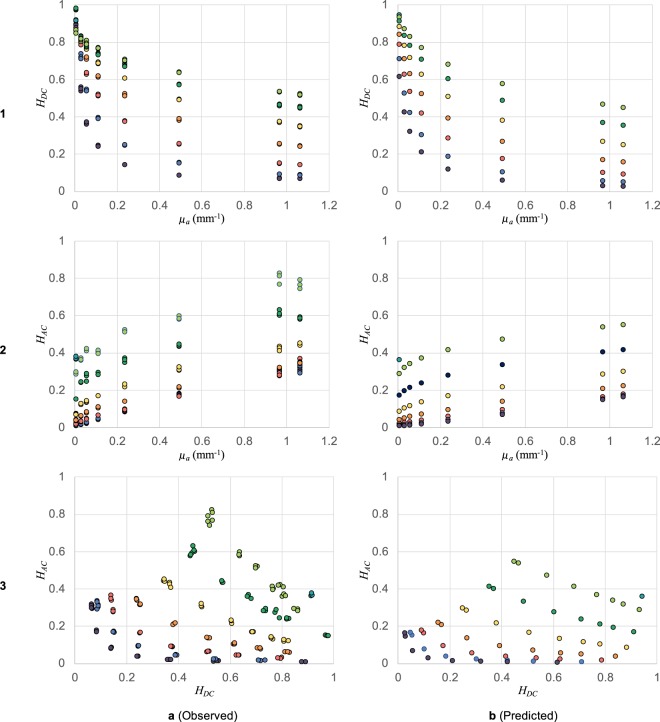
Parameters *H*_*DC*_ and *H*_*AC*_ calculated using eqs (5) and (6) from experimental data and system response model.

We use our set of 57 liquid tissue phantoms to characterize and validate the method. We collected experimental data using the setup described in Fig. 4a and corrected the images to unit exposure time. Cropped backscatter images, original and corrected, for each phantom are included in the accompanying Supplementary Material for reference. We also predicted the model system response for each phantom using WMC simulations for the known reference light transport properties. We then used Equations (5) and (6) to separately reduce the experimental and model data to the two response parameters: DC Response *H*_*DC*_ and AC Response *H*_*AC*_. The observed and predicted parameters are presented for comparison in Fig. 6. We require each (*H*_*DC*_, *H*_*AC*_) pair to have a one-to-one mapping to a unique (*μ*_*a*_, ${\mu ^{\prime} }_{s}$) pair. We also care about the accuracy and the precision with which we can estimate (*H*_*DC*_, *H*_*AC*_) for a given sample. This estimation error combined with the sensitivity of these parameters to changes in (*μ*_*a*_, ${\mu ^{\prime} }_{s}$) eventually determines the instrument’s resolution and error range.

Row 1 of Fig. 6a,b presents the observed and predicted DC response *H*_*DC*_ for all the phantoms. The colour scheme and *μ*_*a*_ axis are the same as those used as in Fig. 4b for clarity. Row 2 of Fig. 6a,b similarly presents the AC response *H*_*AC*_. To observe repeatability, we had collected 3 independent sets of observations for each phantom. Therefore, there are 3 closely-spaced points for each sample in the observed data. The observed and predicted responses closely follow the same trends. However, there are differences in the absolute values in the form of scaling and offset. We can attribute these differences to the assumptions and simplifications in WMC simulations and to the unknown edge artefacts and non-ideal imaging system characteristics of the setup. Fortunately, both scale and offset can be corrected using calibration with standard turbid samples. It is therefore essential to calibrate individual instrument designs and hardware instead of solely relying on model system response simulations.

Row 3 of Fig. 6a,b presents each sample on the *H*_*DC*_ − *H*_*AC*_ plane. One can again observe the differences in predicted and observed data, but calibrate them for a given imaging configuration. More importantly, it is evident that each phantom represents a unique point on the *H*_*DC*_ − *H*_*AC*_ plane, as required. An appropriate spatial frequency content of the projected pattern is essential for a unique mapping along the *H*_*AC*_ axis. Samples with higher *μ*_*a*_ and ${\mu ^{\prime} }_{s}$ have a higher cut-off frequency (and higher *H*_*AC*_), as shown in Fig. 5b. The projected pattern determines the upper limit of observable cut-off frequency, and therefore limits the distinguishable range of (*μ*_*a*_, ${\mu ^{\prime} }_{s}$) pair. Usually it will be preferred to limit the spatial frequency content to the narrowest possible band according to the application. Narrowing the band increases signal energy per unit Δ*k*_*r*_ and therefore decreases *H*_*AC*_ estimation error and increases precision and sensitivity in the samples of interest. Using speckle patterns gives us this flexibility, as we described in Fig. 3.

Another important factor is the dynamic range of the imaging system. It determines the estimation error in the *H*_*DC*_ axis. Phantoms with high *μ*_*a*_ or low ${\mu ^{\prime} }_{s}$ have a weaker backscatter image brightness (and weaker *H*_*DC*_), than others. The points closer to *H*_*DC*_ = 0 are susceptible to image noise and are therefore harder to distinguish. We do not see this happening in Fig. 6a3 expressly because our imaging system adapted exposure to maximize SNR. In practice, we can instead make the illumination system adaptive to preserve the speed of the instrument.

## Discussion

Structured illumination is known for its use in 3-dimensional stereoscopic imaging. It is also used in other nuanced applications such as super-resolution microscopy and diffusion imaging, the latter of which is discussed in this paper. We can use optical elements like micromirror devices and diffraction gratings to generate a range of deterministic structured light patterns ranging from discrete sinusoids to pseudo-random dot patterns. In our research, we are interested in instead using truly-random speckle patterns as structured light for a few reasons. First, speckle patterns are easier and cheaper to generate. Although we currently use a bulky servo-mounted card and focusing optics to generate speckles, it is not hard to imagine it miniaturized to a chip-scale device. Second, the spatial frequency content of speckle patterns is diffraction limited which is much better than the limits afforded by micromirror devices. Further, the frequency band is easily controlled by tuning the aperture and other optical parameters, as we discussed. Finally, independent speckle patterns representing the same random process are easily generated at a high frame rate. Although we discussed using speckle patterns only for diffusion imaging in this paper, we wish to emphasise its general utility as structured illumination in a variety of applications.

Our intention when choosing to use random structured illumination instead of discrete sinusoids is to enable hand-held devices and moving targets. The broadband nature of random structured illumination trades redundancy and accuracy for measurement speed. If the target moves between each frame, we should ideally be able to measure light-transport properties as a time series function. The maximum acceptable mobility of the subject is limited by the image sensor integration time. Image sensors operate at 25 to 125 frames per second (fps), which is faster than typical human movements of up to 10 Hz. On the other hand, if the target is indeed stationary, we can collect multiple observations in rapid succession and reintroduce redundancy and increase accuracy. Eventually, such a system will need to be adaptive to distinguish moving and stationary frames.

Using random or pseudo-random input in system identification presents its merits and demerits. An important limitation is the assumption that the unknown system is linear and invariant. Fortunately, optical diffusion is justifiably assumed as a linear process, ignoring inelastic scattering and interferometric phenomena. However, assuming the system was spatially invariant is not always valid. While we limit our present work to homogeneous tissue phantoms which offer spatial invariance, internal reflections in the cuvette introduced edge effects and belied the assumption. Although we presently eliminate these using cross-correlations, this will not work for skin and other biological media that are inherently heterogeneous. In diagnostic applications, the desire is to observe the heterogeneity instead of obscure it. This is an interesting next step and we leave it for future work.

In summary, using random structured illumination and especially speckle patterns for measuring light transport properties offers its advantages over tradition methods. It is an essential change to present methods if we wish to make portable, simple and fast imaging devices for dermatological applications. We here demonstrated the first essential components of such a system, creating a strong case for further development. Eventually we imagine using a combination of random and deterministic patterns with multiple image sensors for rapid *in-vivo* analysis of skin tissue.

## Supplementary information

Supplementary Material

## Data Availability

The authors confirm that all of the data used in this study are available without restriction. Data can be obtained by contacting sesarma@mit.edu.
