# Supplementary material for: Measuring light transport properties using speckle patterns as structured illumination
Source: Sci Rep. 2019 Aug 1;9:11157. doi: 10.1038/s41598-019-47256-8 (PMC6672017; doi:10.1038/s41598-019-47256-8)
Supplement: Supplementary file 1 — Supplementary Material [file 41598_2019_47256_MOESM1_ESM.pdf]

Measuring light transport properties using speckle patterns as structured illumination

Pranay Jain[1] **and Sanjay E. Sarma**[1,*]

[1]Field Intelligence Laboratory, Department of Mechanical Engineering, Massachusetts Institute of Technology, Cambridge MA 02139, USA
[*]sesarma@mit.edu

Supplementary Material

Fully Developed Speckles as White Noise

Figure S1 illustrates a simple optical configuration to generate objective speckle patterns. A collimated beam of coherent light is incident on a rough surface with a randomly varying surface texture height $f(x',y')$. The scattered light forms the characteristic granular speckles on the parallel screen kept at a distance D. The intensity u of the light pattern at any point (x,y) or $\vec{\rho}$ on the screen is proportional the square of the complex electric field amplitude $\underline{E}$:

$$I(x,y) = I(\vec{\rho}) = \frac{1}{2\eta}\left|\underline{E}(\vec{\rho})\right|^2 \tag{S1}$$

Here, η is the characteristic impedance of the medium, and usually suppressed for brevity. Since singly-scattered light retains its coherence, wavefronts that originate from the many scattering points on the rough surface interfere on the screen. The electric field $\underline{E}(\vec{\rho})$ is therefore expressed as the sum of multiple phasors:

$$\underline{E}(\vec{\rho}) = \int \underline{E}_s(\vec{\rho}',\vec{\rho})\frac{e^{j2\pi r/\lambda}}{r}d\vec{\rho} \tag{S2}$$

Here, $\underline{E}_s(\vec{\rho}',\vec{\rho})$ is the electric field amplitude of the scattered wavefront at a point $\vec{\rho}'$ on the rough surface, towards the point $\vec{\rho}$ on the screen. λ is the wavelength of incident light, r is the scalar distance between points $\vec{\rho}'$ and $\vec{\rho}$. The phasor $\underline{E}_s(\vec{\rho}',\vec{\rho})$ has an additional independent random phase angle proportional to $f(x,y)$ because of the corresponding path length difference. If $f(x,y)$ is of the order of the λ, which is usually the case, electric field $\underline{E}(\vec{\rho})$ is effectively a sum of random phasors.

It is possible to describe the first-order statistics of speckle patterns by analysing sums of random phasors. However, for an intuitive explanation of the second-order statistics, we proceed to simplify the expression in eq. (S2) for far-field speckles using the paraxial approximation and the plane wave assumption. The paraxial approximation $(D \gg |\vec{\rho}' - \vec{\rho}|)$ is used in Fresnel Diffraction Formula for free space propagation. Using it we can approximate distance r and therefore the Electric field $\underline{E}(\vec{\rho})$ as follows:

$$r \approx D - \frac{1}{2D}\left|\vec{\rho}' - \vec{\rho}\right|^2 \tag{S3}$$

$$\underline{E}(\vec{\rho}) \approx e^{j\frac{2\pi|\vec{\rho}|^2}{2\lambda D}}\frac{e^{j2\pi D/\lambda}}{D}\int \underline{E}_s(\vec{\rho}',\vec{\rho})e^{\frac{j2\pi|\vec{\rho}'|^2}{2\lambda D}}e^{\frac{j2\pi\vec{\rho}'\cdot\vec{\rho}}{\lambda D}}d\vec{\rho}' \tag{S4}$$

We rewrite $(\vec{\rho}/\lambda D)$ as spatial frequency $\vec{k}$. This reduces the integral in eq. (S4) to a Fourier Transform:

$$\underline{E}(\vec{\rho}) \approx e^{j\frac{2\pi|\vec{\rho}|^2}{2\lambda D}}\frac{e^{j2\pi D/\lambda}}{D}\mathscr{F}\left[\underline{E}_s(\vec{\rho}',\vec{\rho})e^{\frac{j2\pi|\vec{\rho}'|^2}{2\lambda D}}\right] \tag{S5}$$

The plane wave assumption $(D/|\vec{\rho}' - \vec{\rho}| \gg \pi|\vec{\rho}' - \vec{\rho}|/\lambda)$ is used in far-field Fraunhofer Diffraction to further extend the paraxial approximation. It allows us to ignore the quadratic phase terms in eq. (S5) and hence simplify the expressions for Electric Field $\underline{E}(\vec{\rho})$ and Intensity $u(\vec{\rho})$:

$$\underline{E}(\vec{\rho}) \approx \frac{e^{j2\pi D/\lambda}}{D}\mathscr{F}\left[\underline{E}_s(\vec{\rho}',\vec{\rho})\right] \tag{S6}$$

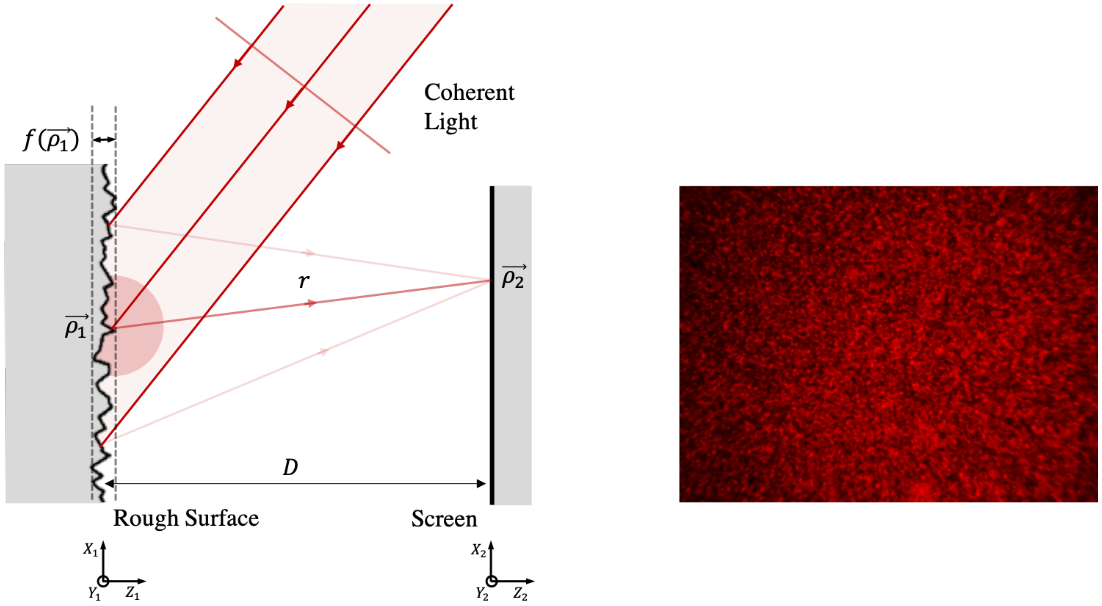

Figure S1. Objective speckles generated due to interference of coherent light reflected of a rough surface

$$u(\vec{\rho}) \approx \frac{1}{2\eta D^2} \left|\mathscr{F}[\underline{E}_s(\vec{\rho}',\vec{\rho})]\right|^2 \tag{S7}$$

The Intensity distribution u on the screen is therefore approximately proportional to the square of the Fourier transform of the scattered electric field $\underline{E}_s$.

We assume that the surface profile $f(x',y')$ is a white noise random process in 2-dimensions. If f is uniformly distributed over a range larger than wavelength λ, phasor angle $\angle\underline{E}_s$ varies uniformly between $-\pi$ and π. Assuming the scattering is isotropic, or the phasor magnitude $|\underline{E}_s|$ is constant, we rewrite the Fourier transform in eq. (S7) as:

$$\mathscr{F}[\underline{E}_s(x',y';x,y)] = |\underline{E}_s| \iint_A e^{j\angle\underline{E}_s(x',y')} e^{-j2\pi\left(x'k_x+y'k_y\right)} dx'dy' \tag{S8}$$

$$\left|\mathscr{F}[\underline{E}_s(x',y';x,y)]\right|^2 = 2|\underline{E}_s|^2 \left[\iint_A \cos\left(\angle\underline{E}_s(x',y')\right) e^{-j2\pi\left(x'k_x+y'k_y\right)} dx'dy'\right]^2 \tag{S9}$$

Here A is the area of the illuminated region on scattering surface. The cosine of a random variable is another random variable, varying between -1 and 1. In eq. (S9), $\angle\underline{E}_s(x',y')$ is a uniform white noise random process, and it's cosine is another such process with a non-uniform distribution. Regardless of the distribution, the Fourier transform of a random cosine function is random itself. This is illustrated in Figures S2a to S2e with the example of a discrete function $x[n] = cos(\phi[n])$, where $\phi[n]$ is a random variable uniformly distributed between $-\pi$ and π, independent of n. The square of the Fourier transform $|X[\Omega]|^2$ is a white noise random number with a χ^2 distribution with 2 degrees of freedom. Similarly, the square of the 2D Fourier transform in eq. (S9) also results in a white noise random variable with a χ^2 distribution with 2 degrees of freedom. The speckle pattern, given the above assumptions is therefore a white noise pattern with a vanishing specular field. The size of individual grains is diffraction limited to wavelength λ. Such speckles are known as *Fully Developed Speckle Patterns*.

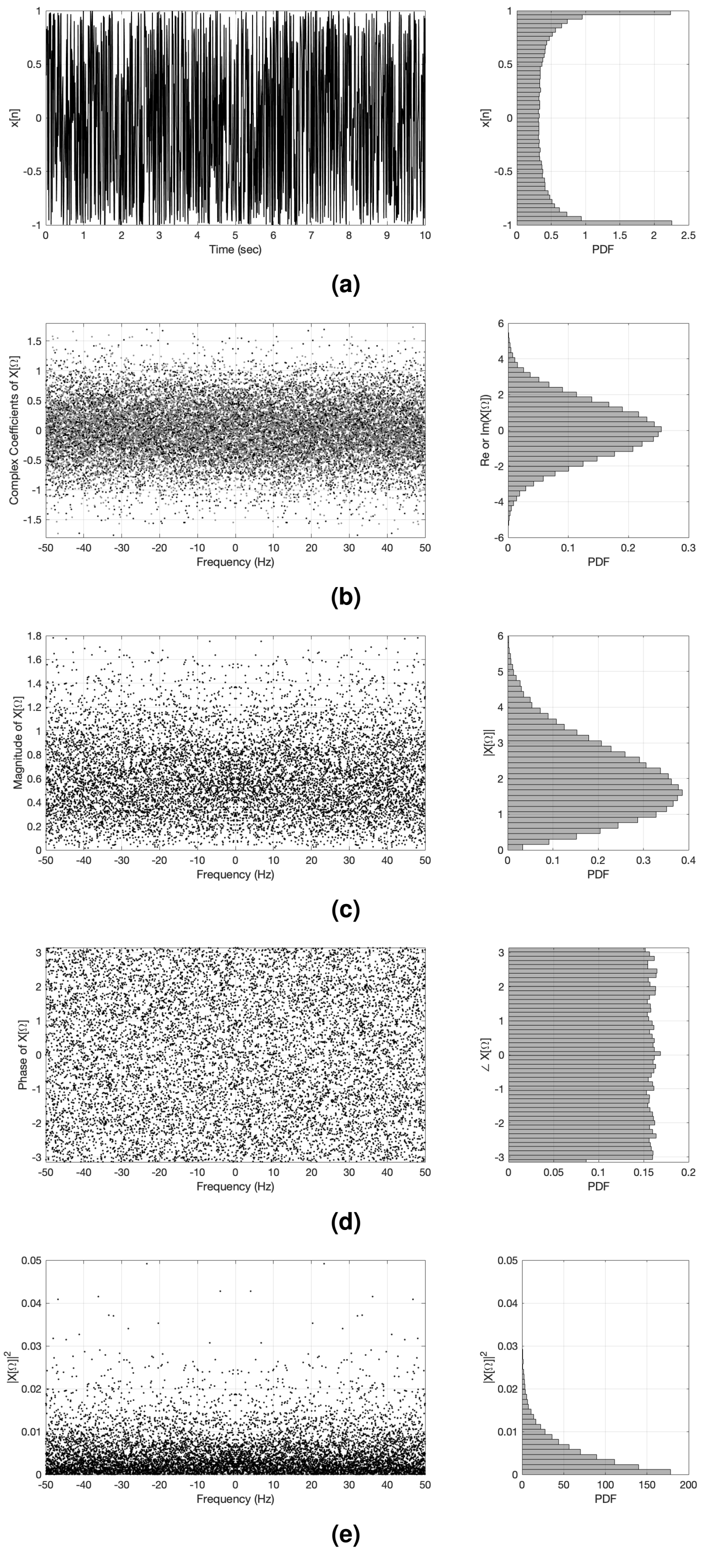

Figure S2. Power spectrum of the cosine of a uniform random variable. (**a**) Probability Density Function (PDF) of the cosine function, (**b**) Combined PDF of the Real and Imaginary parts of the Fourier Transform, (**c**) PDF of the Fourier Transform magnitude, (**d**) PDF of the Fourier Transform angle, and (**e**) PDF of the Power Spectrum

Stationarity and Ergodicity of Speckle Patterns

In the present work, we are interpreting random speckle patterns as broadband spatial input for system identification. This relies on the assumption that input speckle patterns are stationary and ergodic. To experimentally validate these assumptions, we collected sets of $N = 10$ independent speckle pattern images for three different aperture sizes. Each optical configuration represents a random process $U(x,y)$ and each image is a finite area realization $u_i(x,y);\ i = 1 \dots N$ of this random process. A cropped portion of one observed speckle pattern for each configuration is shown in Figures S3a to S3c (1) for illustration. To experimentally characterize a random process is challenging with finite data. Therefore, we limit the scope to validating that the process is Wide-Sense Stationary (WSS) and ergodic in the mean. We first discuss the stationarity because ergodicity is not defined for non-stationary processes.

For a time-series WSS process, the first and second order statistics remain invariant with time. A necessary and sufficient condition for WSS stationarity is that the Auto-Correlation Function (ACF) is dependent only on time delay and independent of position in time. We extend this to 2D processes in XY space. Figures S3a to S3c (2) give the estimated ACF $\hat{R}_{UU}(\Delta x, \Delta y)$ of $U(x,y)$ for each configuration. We arrive at the estimate by averaging the biased ACF estimates for all $u_i(x,y)$ realizations:

$$\hat{R}_{UU}(\Delta x, \Delta y) = \frac{1}{N} \sum_{i=1}^{N} \hat{R}_{u_i u_i}(\Delta x, \Delta y) \tag{S10}$$

$$\hat{R}_{u_i u_i}(\Delta x, \Delta y) = \frac{1}{PQ} \sum_{p=1}^{P-|\Delta x|} \sum_{q=1}^{Q-|\Delta x|} u_i[p+\Delta x, q+\Delta y] \cdot u_i^*[p,q] \tag{S11}$$

Here $P = 1280$ and $Q = 1024$ are the number of pixels captured per realization along X and Y directions. The ACF estimate has elliptical contours because of the elliptical shape of the aperture. For clarity, Figures S3a to S3c (3) give the individual and averaged ACF along the X direction ($\Delta y = 0$). Figures S3a to S3c (4) give the same along the Y direction ($\Delta x = 0$). Since the ACF estimate decays much faster than the maximum observed spatial delay, we can confidently claim that the process is WSS. Note that the images were mean-centred and normalized before estimating the ACFs, hence auto-correlation is the same as auto-covariance.

A time-series process is ergodic in the mean if the average along time *almost always* equals the average across multiple realizations. In essence, it ascertains that each independent realization of a stationary random process is statistically identical. Figures S3a to S3c (5) give the cross-correlations $\hat{R}_{u_i u_j}(\Delta x, \Delta y);\ i \neq j$, averaged for all ${}^{N}\mathbf{C}_2 = 45$ possible pairs of realizations. Since the cross-correlation is always below 5×10^{-3}, it is statistically insignificant. Therefore, we can consider each realization independent of all others. Figures S3a to S3c (6) gives a histogram of the means of pixel readings across realizations and the mean estimated across XY space for each realization. The average mean estimate is close to the estimate from each realization. Figures S3a to S3c (6) gives a histogram of the standard deviations of pixel readings across realizations and that estimated across XY space for each realization. We arrive at the same conclusion as earlier. We can therefore confidently claim that the process is ergodic in the mean.

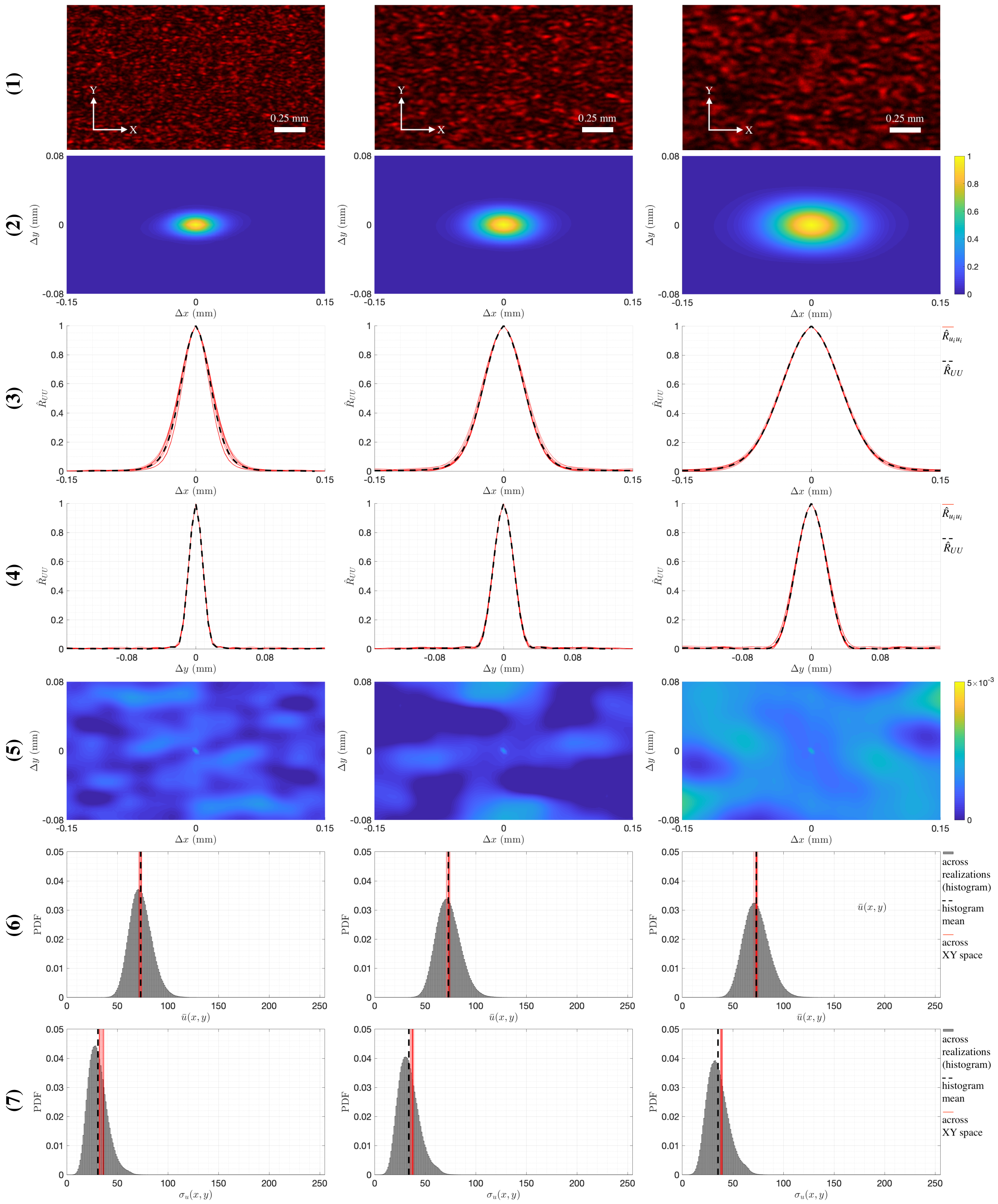

(a) 3.5mm×1.9mm **(b)** 2.5mm×1.3mm **(c)** 1.5mm×0.8mm

Figure S3. Statistics of speckle patterns observed at three (**a** to **c**) elliptical aperture sizes: (**1**) Sample observations. (**2**) Estimated ACF $\hat{R}_{UU}(\Delta x, \Delta y)$, (**3**) $\hat{R}_{UU}(\Delta x, 0)$, (**4**) $\hat{R}_{UU}(0, \Delta y)$. (**5**) Average estimated cross-correlation $\hat{R}_{u_i u_j}(\Delta x, \Delta y)$. (**6**) Mean and (**7**) standard deviation of pixel readings across space versus across XY space.

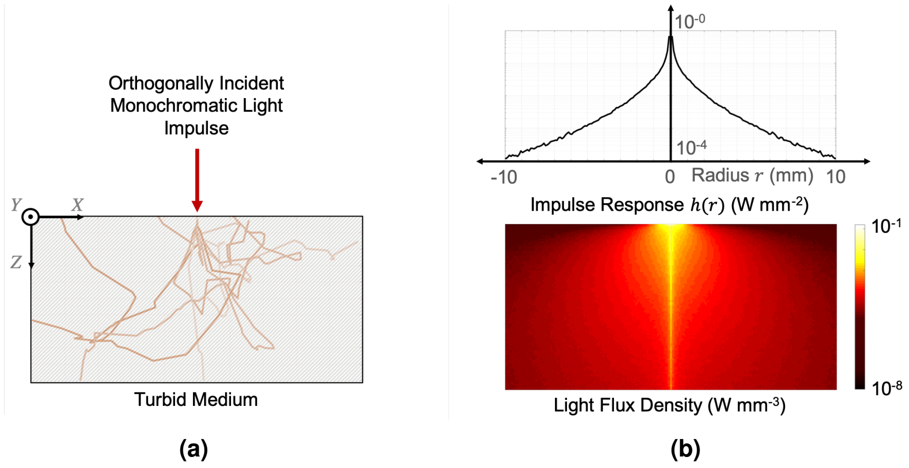

Figure S4. Sample results from MC Ray Tracing: (**a**) Random paths for 10 randomized photon scattering simulations, (**b**) Light flux density in the semi-infinite medium and the radially-symmetric backscatter impulse response $h(r)$, predicted using 100,000 photon scattering simulations

Backscatter Impulse and Frequency Response

When a narrow beam of light with unit power is incident at any point (x_0, y_0) on a turbid medium, the diffuse backscatter is the linear 2D impulse response $h(x, y; x_0, y_0)$. For a uniform semi-infinite medium, the impulse response is independent of position (x_0, y_0) and reduces to a radially symmetric function $h(r)$, where r is the scalar distance from point (x_0, y_0). We estimate the impulse response $h(r)$ for a given turbid medium with known light transport properties using White Monte Carlo (WMC) Ray Tracing simulations. The results for one such simulation are shown in Figure S4 for illustration.

Next, we define the spatial frequency response $H(k_x, k_y)$ as the Fourier transform of 2D impulse response $h(x, y)$. Since $h(x, y)$ reduces to a radially symmetric function $h(r)$, the 2D Fourier transform also reduces to a zeroth-order 1D Hankel transform. Figure S5 presents the impulse and frequency responses estimated for a range of light transport properties (μ_a, μ_s' and g).

$$H(k_x, k_y) = \mathscr{F}\left(h(x, y)\right) = \iint h(x, y) e^{-jk_x x} e^{-jk_y y} dx dy \tag{S12}$$

$$\begin{aligned} H(k_r) = \mathscr{H}_0\left(h(r)\right) &= \int_r \left[h(r) \int_0^{2\pi} \left(e^{j2\pi k_r r \cos\theta} d\theta \right) r dr \right] \\ &= 2\pi \int_r \left[h(r) J_0(k_r r) r dr \right] \end{aligned} \tag{S13}$$

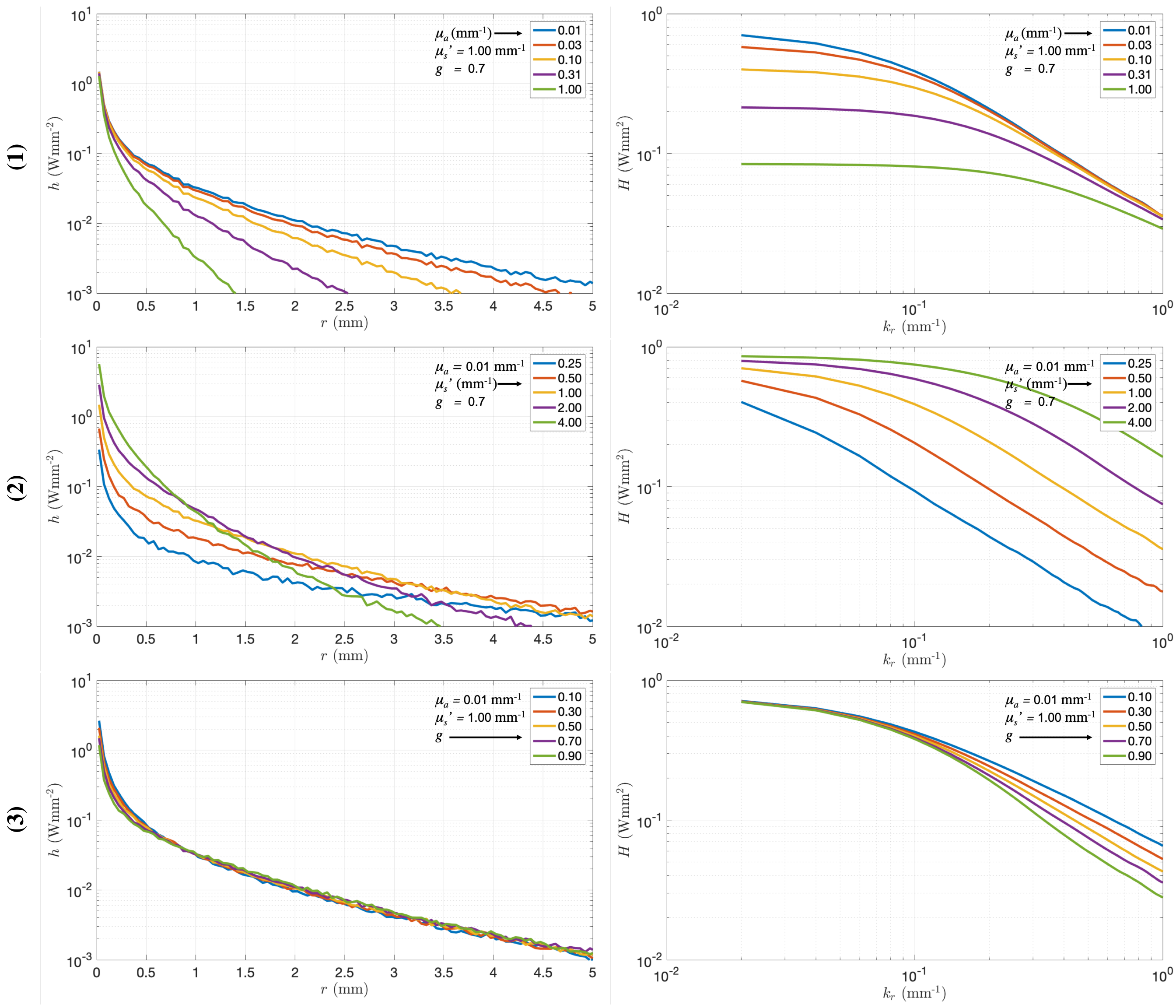

(a) Impulse Response $h(r)$ **(b)** Frequency Response $H(k_r)$

Figure S5. Predicted backscatter response for a range of light transport properties: (**1**) $\mu_a \in [0.01, 1.00]$ mm-1, $\mu_s' = 1$mm-1 and $g = 0.7$, (**2**) $\mu_a = 0.01$mm-1, $\mu_s' \in [0.25, 4.00]$ mm-1 and $g = 0.7$, and (**3**) $\mu_a = 0.01$mm-1, $\mu_s' = 1.00$mm-1 and $g \in [0.1, 0.9]$

Backscatter Sample Images

Figures S6a and S6b presents a cropped backscatter image for each liquid tissue phantom. Backscatter images are cropped to a 100×100 pixel^2 or 5×5 mm^2 area. Figure S6a presents the images as captured by the camera with automatic exposure control. This lets us observe the spatial detail in the images, or simply the AC component, even for dark samples with high μ_a. Figure S6b presents the same images, corrected for unit exposure. The details are hidden for darker samples, as expected.

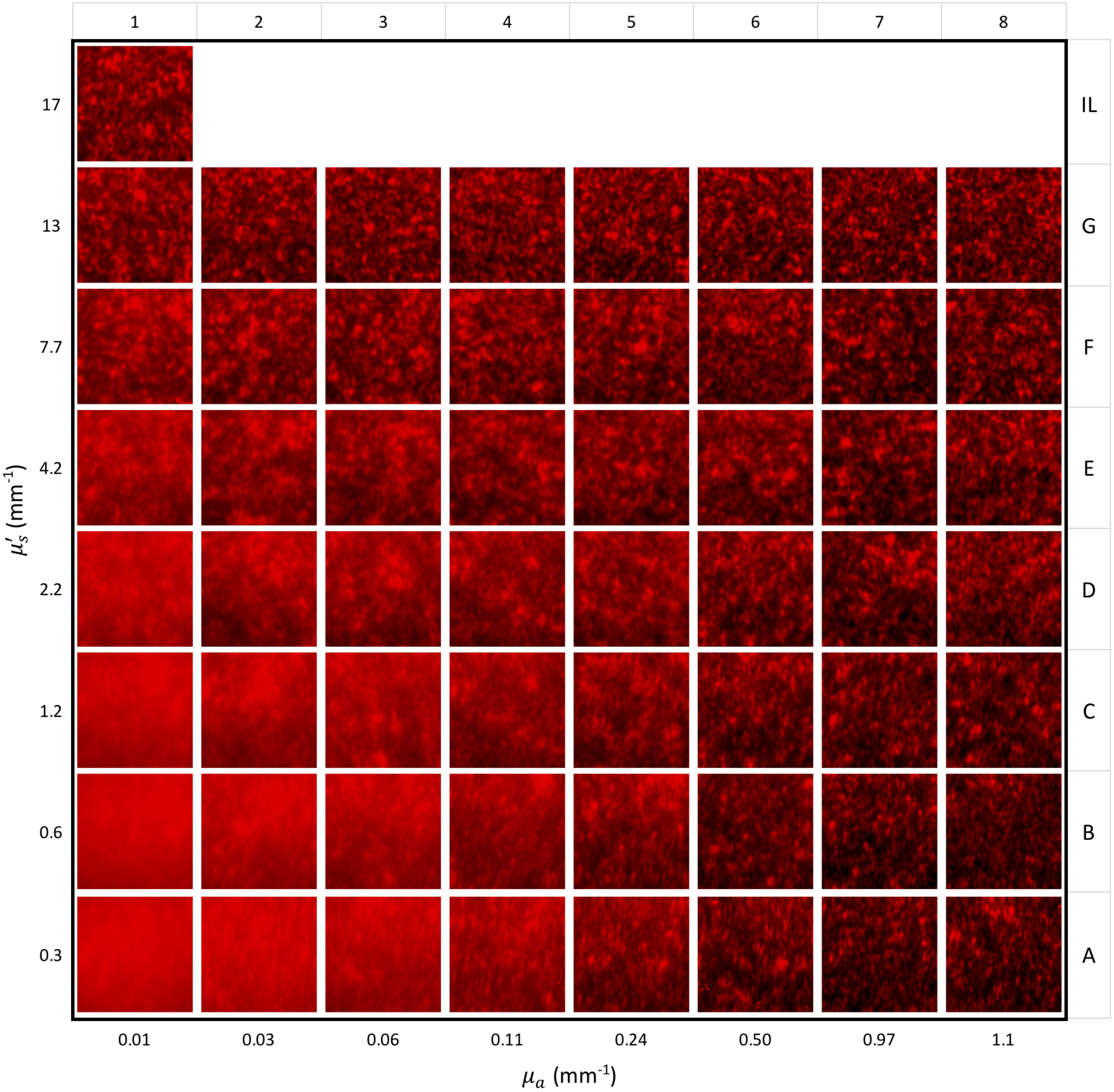

(a)

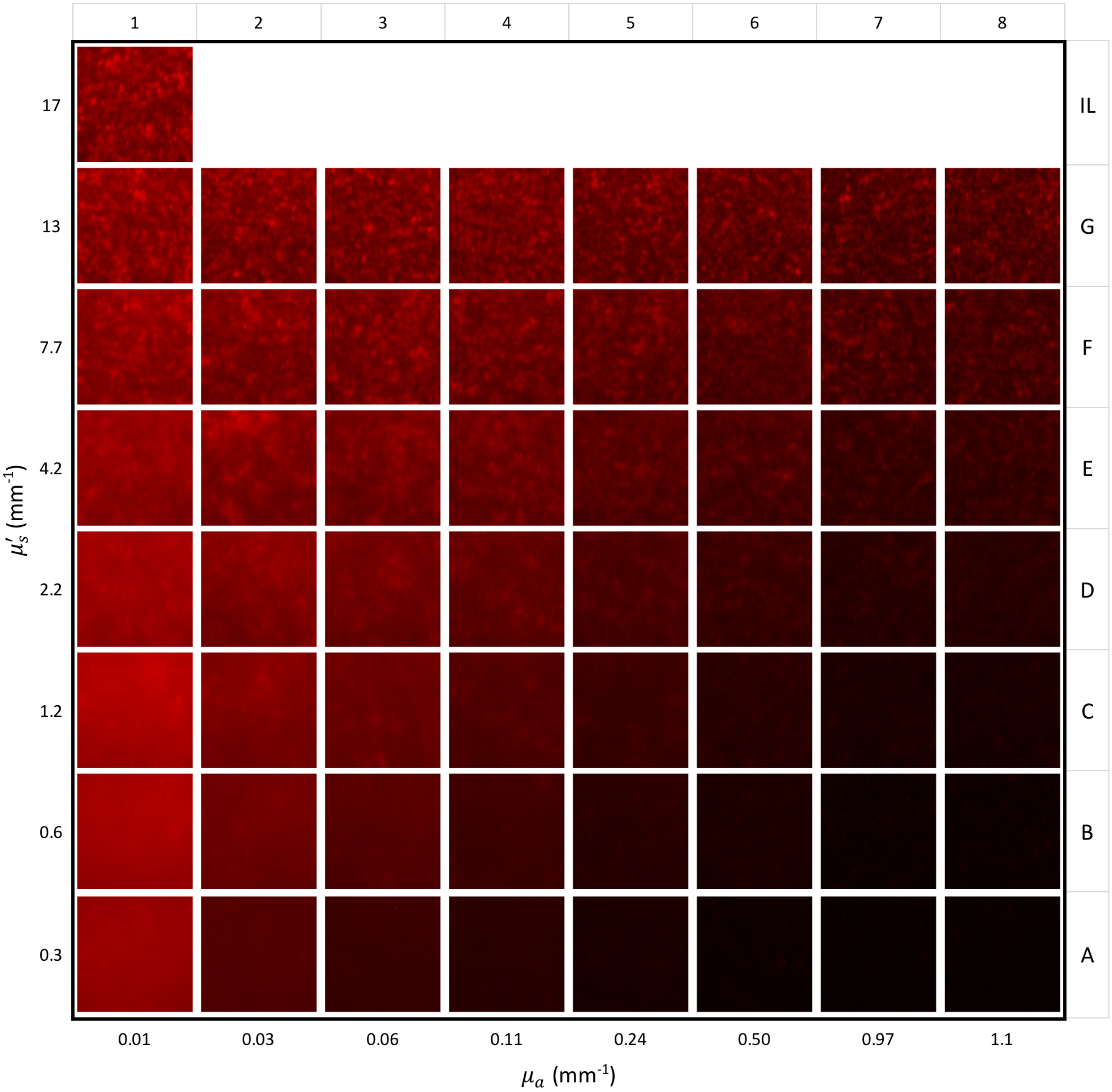

(b)

Figure S6. Backscatter images observed for each liquid tissue phantom, cropped to a 100×100 pixel^2 or 5×5 mm^2 area: **(a)** As captured by the camera with automatic exposure control, **(b)** Corrected to unit exposure
